# Supplementary material for: Fluorescent Biosensors for Cryogenic Correlative Light and Electron Microscopy
Source: bioRxiv. 2026 Oct 19:2026.10.15.759416. Preprint. [Version 1] doi: 10.64898/2026.10.15.759416 (PMC13738430; doi:10.64898/2026.10.15.759416)
Supplement: Supplementary file 1 [file media-1.pdf]

Supplementary Information

Fluorescent Biosensors for Cryogenic Correlative Light and Electron Microscopy

Christopher A. Azaldegui[1,2], Kevin M. Rose[1,2], Davis Perez[3], Jacob A. Summers[1], Claudia A. Mak[4], William P. Dwyer[5], Anthony G. Vecchiarelli[6], Julie S. Biteen[7], José R. Dinneny[5], and Peter D. Dahlberg[1,2*]

[1] Department of Structural Biology, Stanford University, Stanford, CA 94305, USA

[2] Division of Cryo-EM and Bioimaging, SLAC National Accelerator Laboratory, Menlo Park, CA 94025

[3] Department of Chemistry, Stanford University, Stanford, CA 94305, USA

[4] Department of Biological Chemistry, University of Michigan Medicine, Ann Arbor, MI 48109, USA

[5] Department of Biology, Stanford University, Stanford, CA 94305, USA

[6] Department of Molecular, Cell, and Developmental Biology, University of Michigan, Ann Arbor, MI 48109, USA

[7] Department of Chemistry, University of Michigan, Ann Arbor, MI 48109, USA

*corresponding author: pdahlb@stanford.edu

Supplementary Table 1. Plasmids used in this study

Plasmid	Description	Source
pDEST-HisMBP-SED1	Bacterial expression of SED1	Addgene #178191, [1]
pRsetB-pHRed	Bacterial expression of pHRed	Addgene #31472, [2]
RpHluorin2	Bacterial expression of pHluorin2	Addgene #180227, [3]
pBAD-pHScarlet	Bacterial expression of pHmScarlet	Addgene #191258
pET15b-eGO-Matryoshka-LE-LP	Bacterial expression of MatryoshCaMP6s	Addgene #100017, [4]
pTorPE-jREX-GECO1	Bacterial expression of jREX-GECO1	Addgene #113941, [5]
pLV-JREX-GECO1-IRES-puro	For stable expression of jREX-GECO1 into U2OS cells	This study
pCMV-VSV-G	For transfection of jREX-GECO1 gene into U2OS cells	Addgene #8454, [6]
cbbS-pHmScarlet	For chromosomal replacement of the native *cbbS* gene in *H. neapolitanus*	This study

Supplementary Table 2. Strains used in this study

Strain	Description	Source
S. cerevisiae (SED1)	*S. cerevisiae* (BY4742) with *pDRFLIP38-SED1*	[1]
AGV_Hn_1	*H. neapolitanus*	ATCC
AGV_Hn_547	*H. neapolitanus cbbS::cbbS-pHmScarlet*	This study

Supplementary Table 3. Fit parameters for biosensor calibrations

Biosensor	Figure	L	k	x_0	b
SED1	Main. 2c	4.35	0.59	8.47	2.73
pHRed	SI. 5b	9.04	1.27	8.17	1.6
pHmScarlet	Main. 2i	102.13	-2.93	6.54	1.48
pHmScarlet	SI. 8a	5.72	-2.93	6.54	0.04
jREX-GECO1	Main. 2f	2.4	2	0.15	-1.07
jREX-GECO1	SI. 7a	1.68	1.34	0.97	0

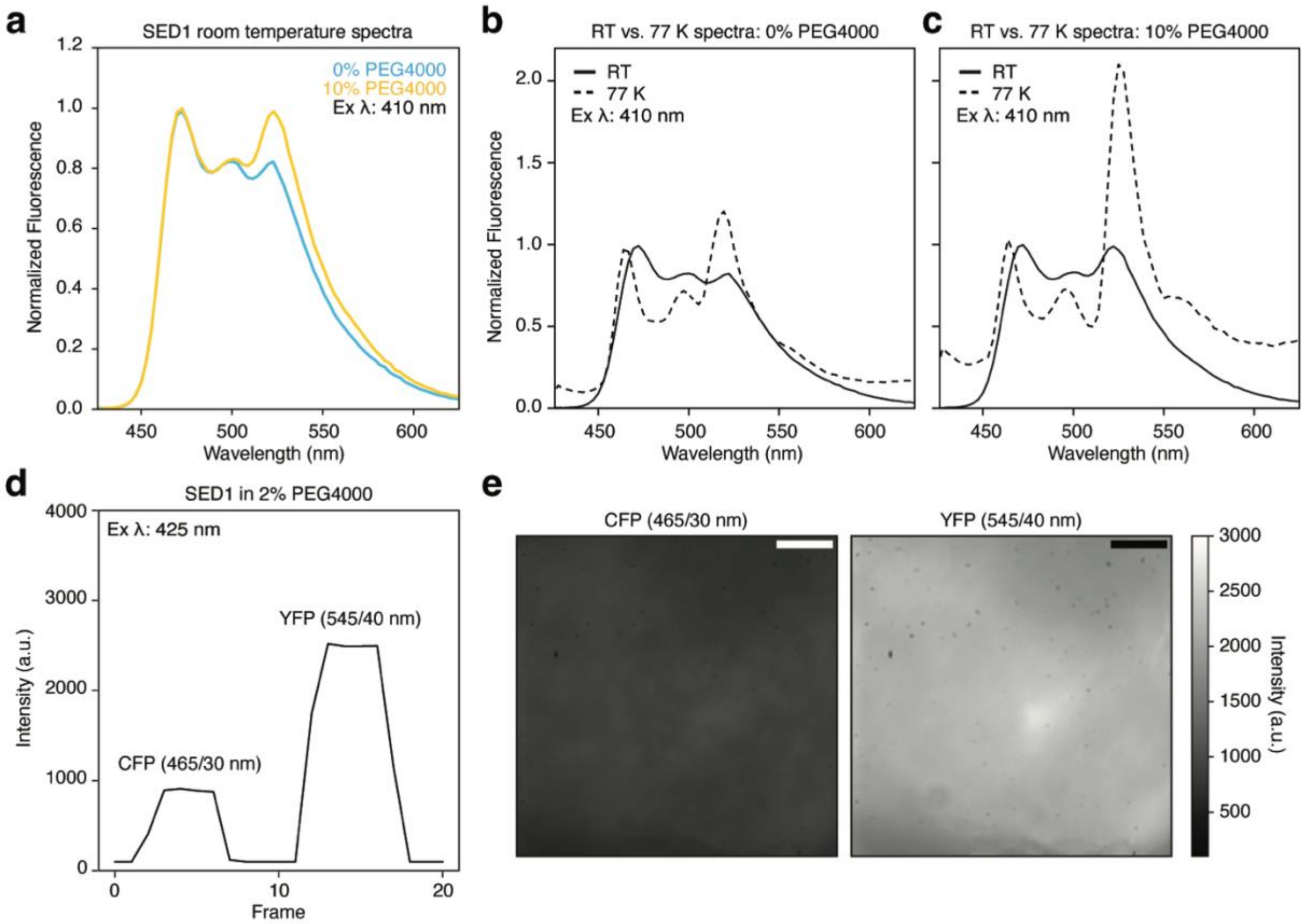

Supplementary Figure 1: *In vitro* characterization of SED1.

a) Fluorescence emission spectra of SED1 in the presence (yellow) and absence (blue) of 10% PEG4000 (w/v) at room temperature (RT). **b)** Fluorescence emission spectra of SED1 in 0% PEG4000 at RT (solid) and 77 K (dashed). **c)** Fluorescence emission spectra of SED1 in 10% PEG4000 at RT (solid) and 77 K (dashed). Emission spectra are normalized to their respective mCerulean peak at 465 nm. **d)** Representative fluorescence intensity trace of high-pressure frozen SED1 in 2% PEG4000 using CFP (465/30 nm) and YFP (545/40 nm) emission filters. **e)** Representative average fluorescent images of high-pressure frozen SED1 in 2% PEG4000. Scale bars: 15 μm.

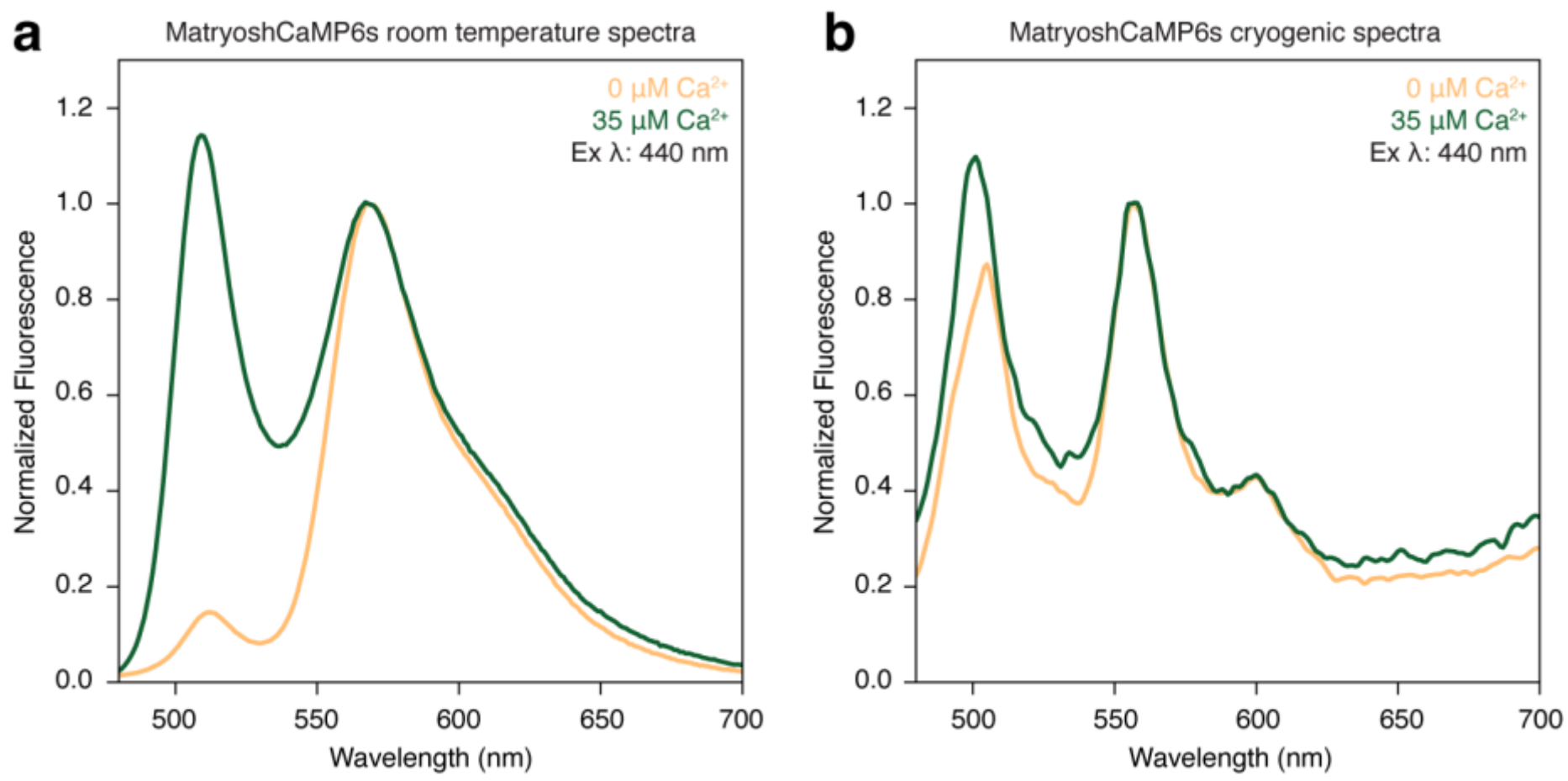

Supplementary Figure 2: Emission spectra of MatryoshCaMP6s at room and cryogenic temperatures.
a) Fluorescence emission spectra of MatryoshCaMP6s in the presence and absence of 35 µM Ca^{2+} at room temperature. Emission spectra are normalized to their respective LSS-mOrange peak at 570 nm. **b)** As in **a**, but at 77 K.

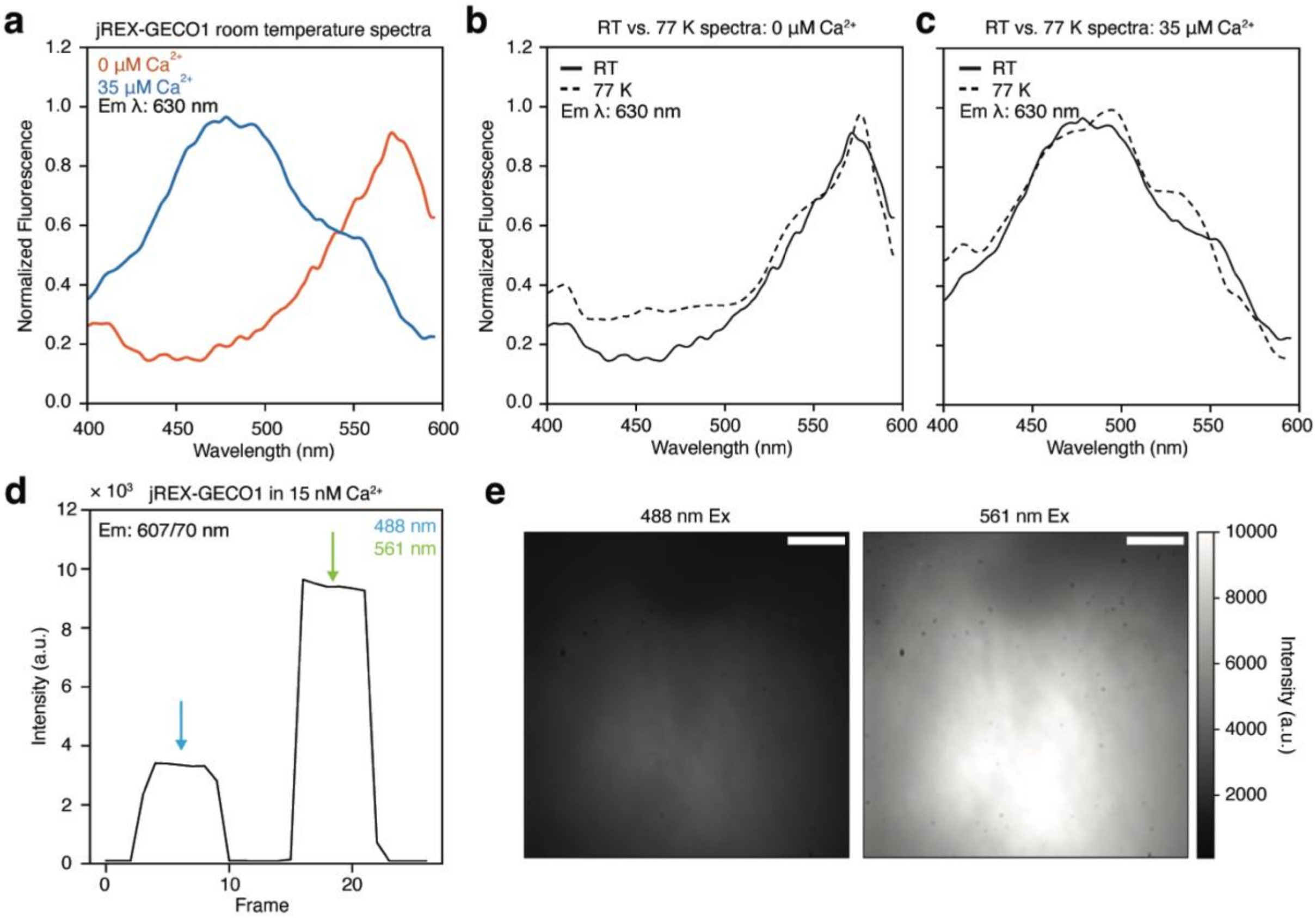

Supplementary Figure 3: *In vitro* characterization of jREX-GECO1. a) Room-temperature excitation spectra of jREX-GECO1 in the presence and absence of Ca^{2+} (35 µM). **b)** Fluorescence excitation spectra of jREX-GECO1 in the absence of Ca^{2+} at RT (solid) and 77 K (dashed). **c)** Fluorescence excitation spectra of jREX-GECO1 in the presence of 35 µM Ca^{2+} at RT (solid) and 77 K (dashed). Excitation spectra are normalized independently. **d)** Representative fluorescence intensity trace of high-pressure frozen jREX-GECO1 in 0.015 nM Ca^{2+} using 488 nm and 561 nm excitation lasers. **e)** Representative average fluorescent images of high-pressure frozen jREX-GECO1 in 15 nM Ca^{2}. Scale bars: 15 µm.

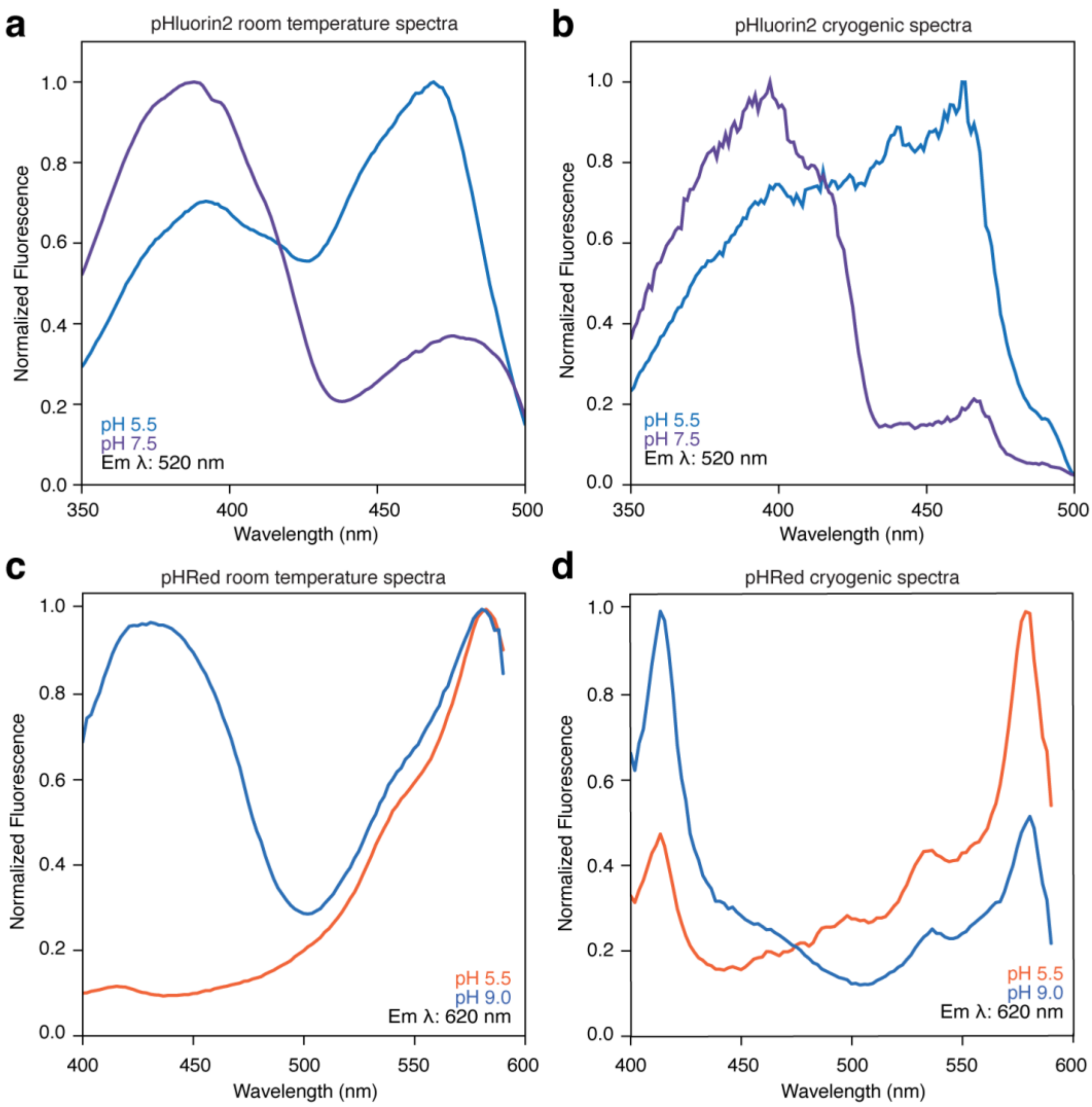

Supplementary Figure 4: Fluorescence spectra of pH sensors at room and cryogenic temperatures. a) Room-temperature excitation spectra of pHluorin2 at pH 5.5 and pH 7.5. **b)** As in **a**, but at 77 K. **c)** Room-temperature excitation spectra of pHRed at pH 5.5 and pH 9.0. **d)** As in **c**, but at 77 K. Excitation spectra are normalized independently.

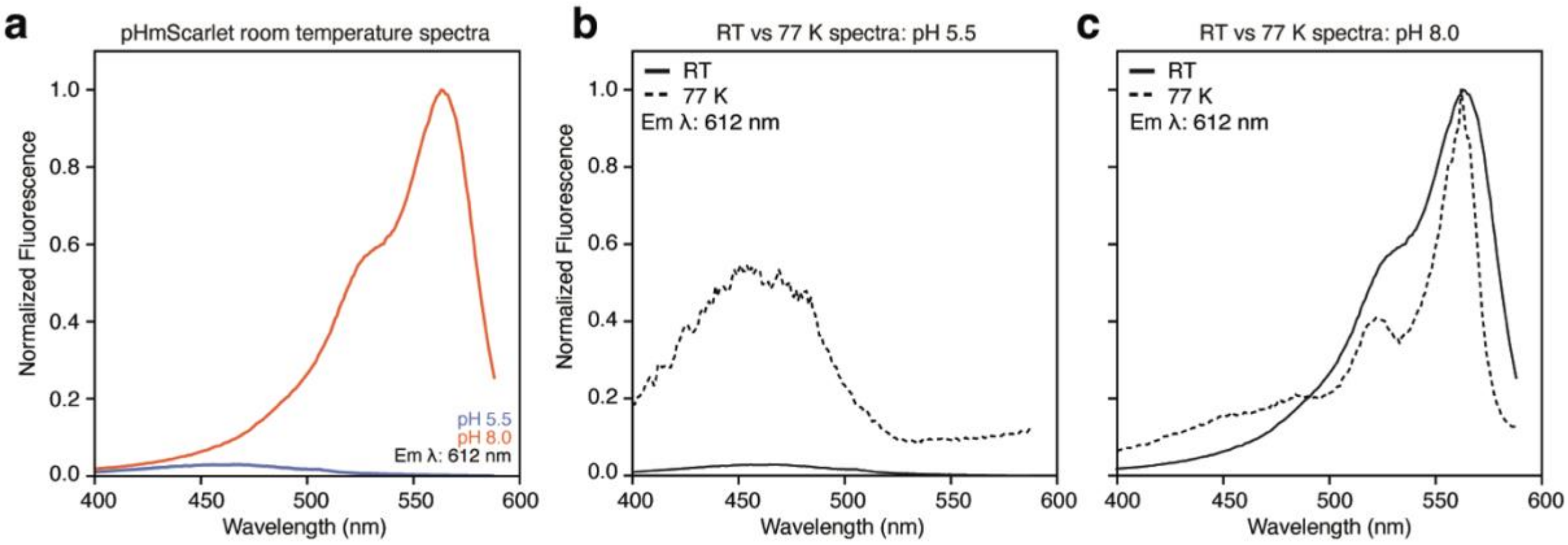

Supplementary Figure 5: *In vitro* characterization of pHmScarlet. a) Room-temperature excitation spectra of pHmScarlet at pH 5.5 (blue) and pH 8.0 (orange). **b)** Fluorescence excitation spectra of pHmScarlet at pH 5.5 at RT (solid) and 77 K (dashed). **c)** Fluorescence excitation spectra of pHmScarlet at pH 8.0 at RT (solid) and 77 K (dashed). Excitation spectra are normalized to the maximum of the corresponding pH 8 spectrum.

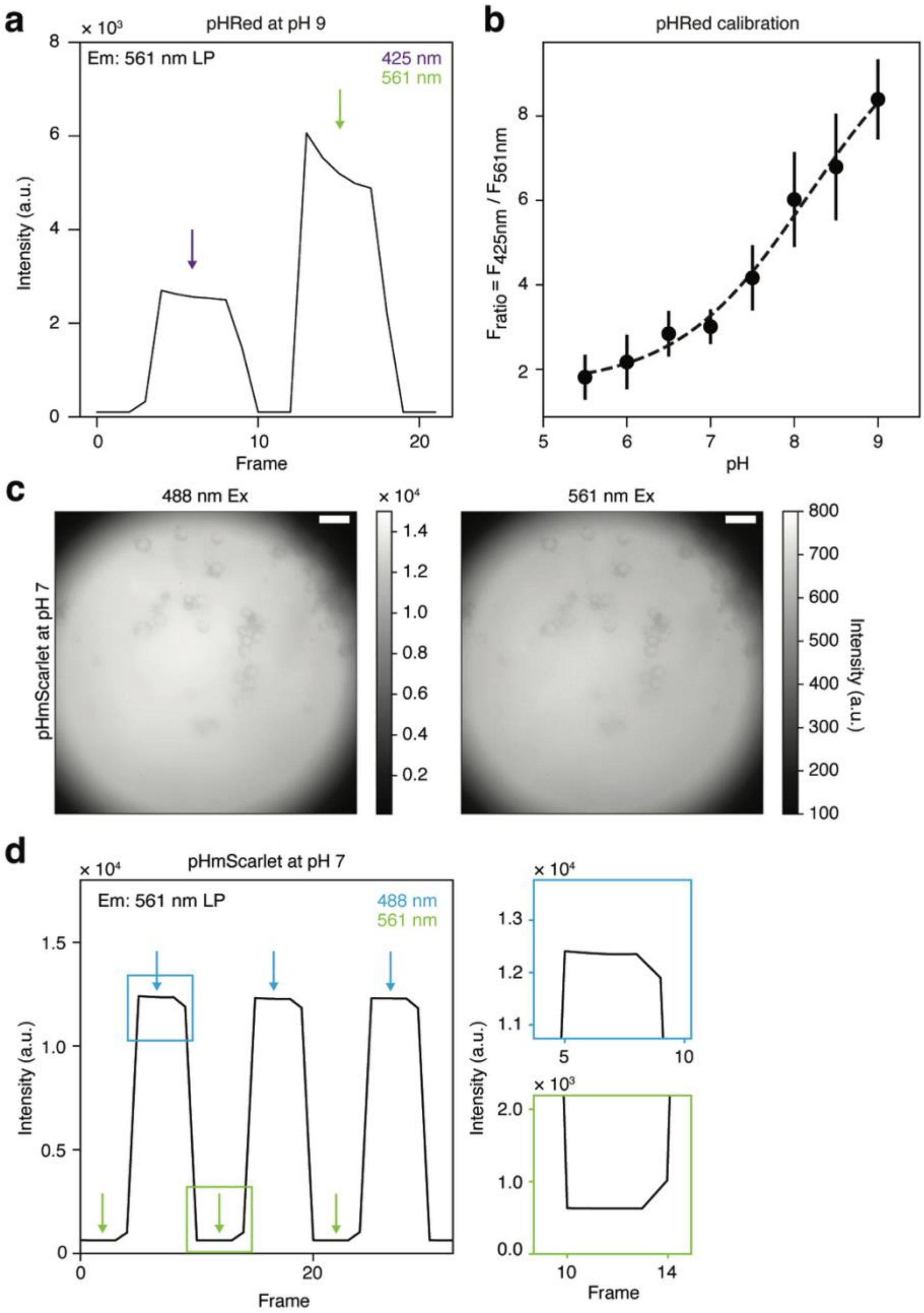

Supplementary Figure 6: pHRed intensity trace and calibration.
a) Fluorescence intensity trace of high-pressure frozen pHRed at pH 9. Intensity peaks are labeled with their respective laser excitation wavelengths. 561 nm excitation results in rapid photobleaching of pHRed. **b)** F_{ratio} for 425- and 561-nm excitation of high-pressure frozen pHRed as a function of pH. Points and error bars represent the average and standard deviation of at least 5 ratiometric measurements for each condition. The dashed line is the fit to a continuous logistic function. **c)** Representative average fluorescent images of high-pressure frozen pHmScarlet in pH 7.0. Scale bars: 25 µm. **d)** Representative fluorescence intensity trace of high-pressure frozen pHmScarlet at pH 7 using 488 nm and 561 nm excitation lasers. Blue inset is zoom into 488 nm excitation trace. Green inset is zoom into 561 nm excitation trace.

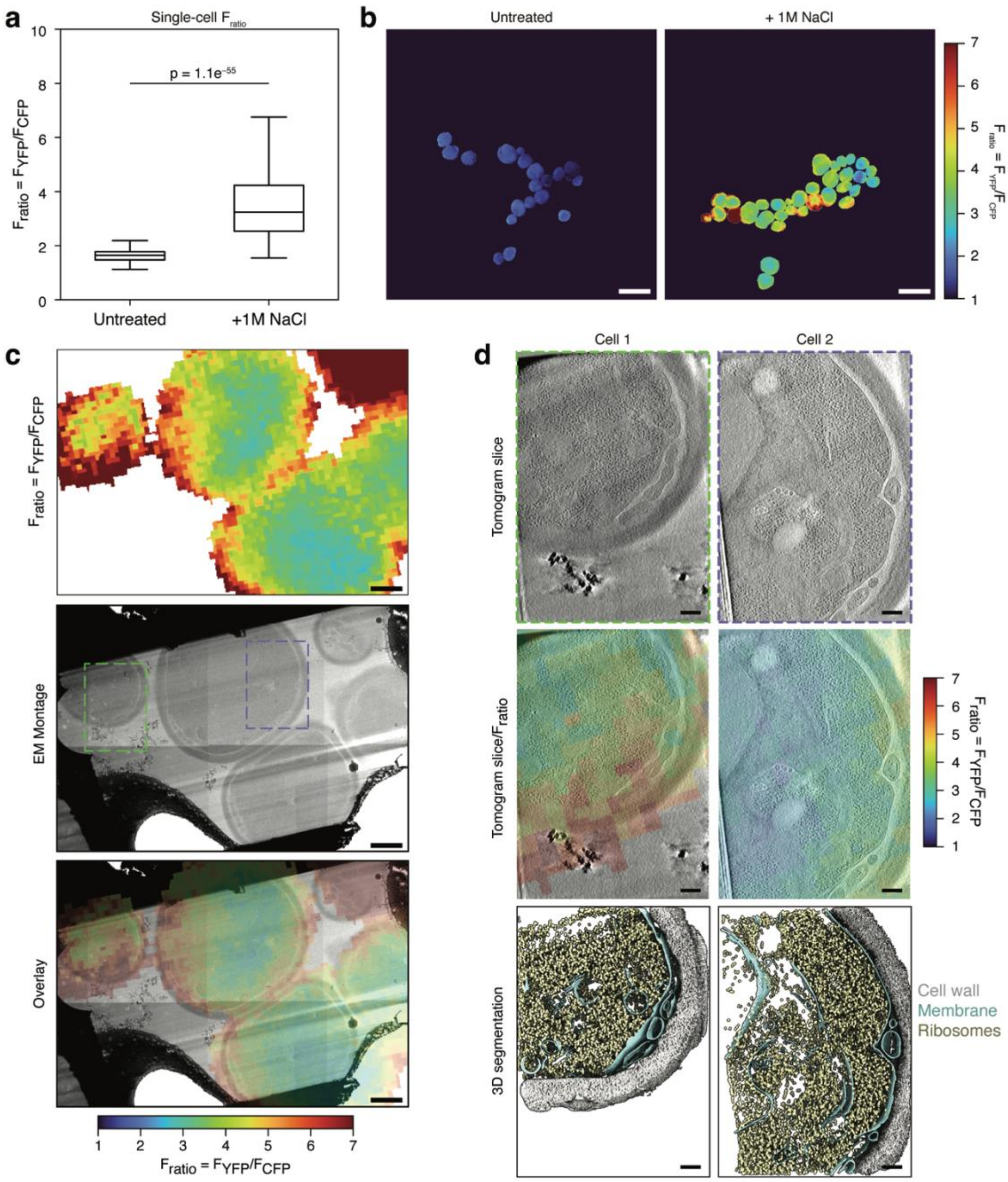

Supplementary Figure 7: CryoCLEM of SED1 in *S. cerevisiae*

a) Single-cell average F_{ratio} with (N = 267 cells) and without (N = 55 cells) osmotic shock. **b)** F_{ratio} (YFP/CFP) fluorescence images of cells with and without osmotic shock. Scale bars: 10 µm. **c)** The top panel is a registered F_{ratio} image corresponding to an EM montage of a lamella of osmotically shocked cells (middle panel). The bottom panel shows the overlay of the montage and the F_{ratio} image. Colored dashed boxes are the tomogram collection regions shown in d. Scale bars: 1 µm. **d)** The top row shows slices from the reconstructed tomograms of the regions shown in **c**. The middle row shows the tomogram slices overlaid with the F_{ratio} image. The bottom row shows the segmentation of ribosomes (gold), membrane (teal), and cell wall (gray). Scale bars: 200 nm.

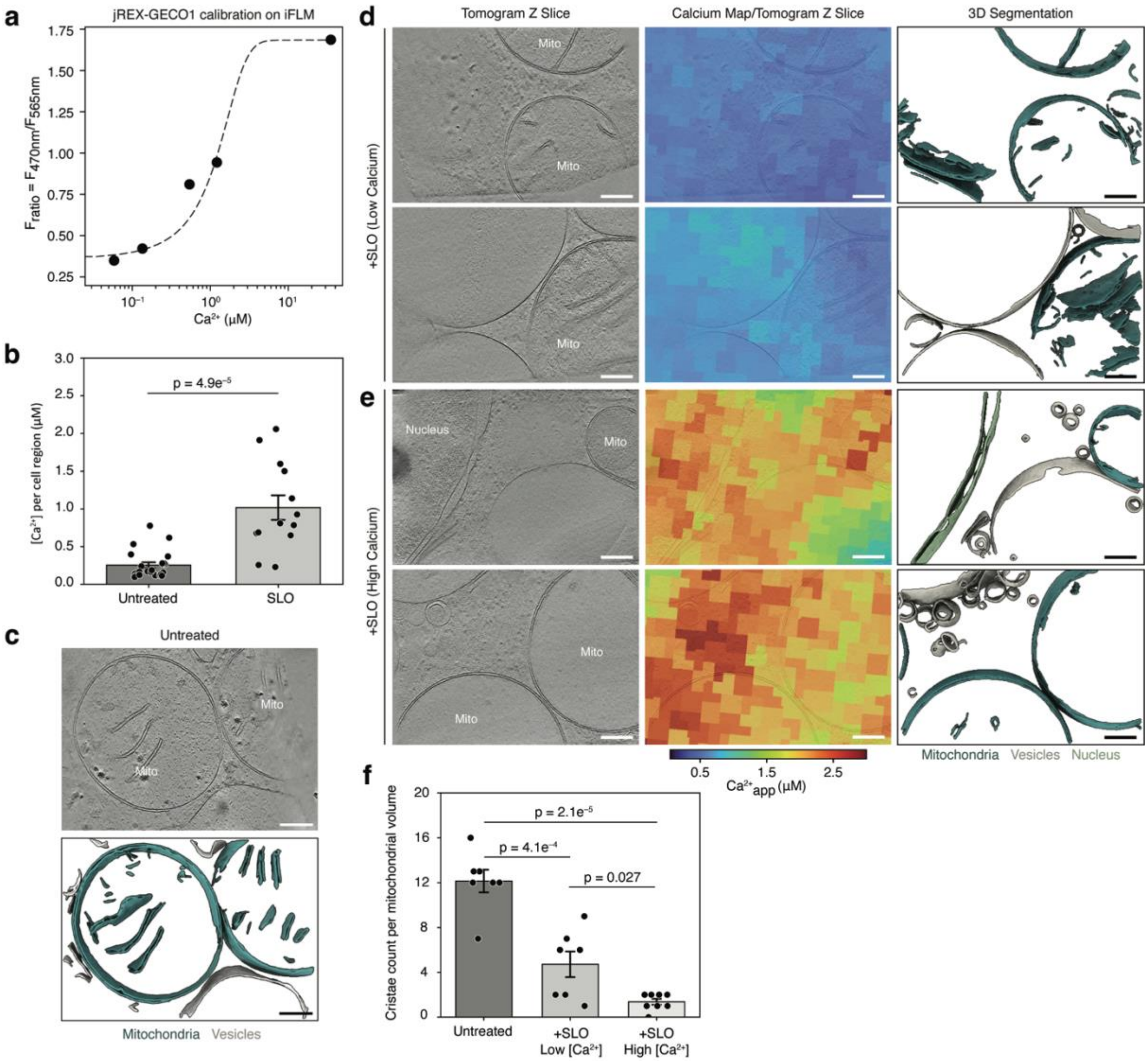

Supplementary Figure 8: CryoCLEM of jREX-GECO1 in U2OS cells.

a) F_{ratio} for 470- and 565-nm excitation of high-pressure frozen jREX-GECO1 as a function of Ca^{2+} concentration. The dataset was collected on the iFLM within an Aquilos2 system. The dashed line is the fit to a continuous logistic function. **b)** Average Ca^{2+} concentration for untreated (*N* = 22 regions) and SLO-treated (N = 13 regions) U2OS cells. **c)** The top panel shows a z-slice from the reconstructed tomogram of an untreated U2OS cell. The bottom panel shows the segmentation of mitochondria (teal) and vesicles (gray). **d)** Two reconstructed tomogram z-slices are shown for SLO-treated U2OS cells with a low measured calcium level. The middle panels show the corresponding tomogram z-slices overlaid with the correlated calcium map. The right panels show the segmentation of mitochondria (teal), vesicles (gray), and the nuclear membrane (green). **e)** As in **d**, but for a cell with a high measured calcium level. Scale bars: 200 nm. **f)** Mitochondrial cristae counts categorized by sample and Ca^{2+} concentration. *N* = 7 mitochondria from 4 to 7 tomograms for each group.

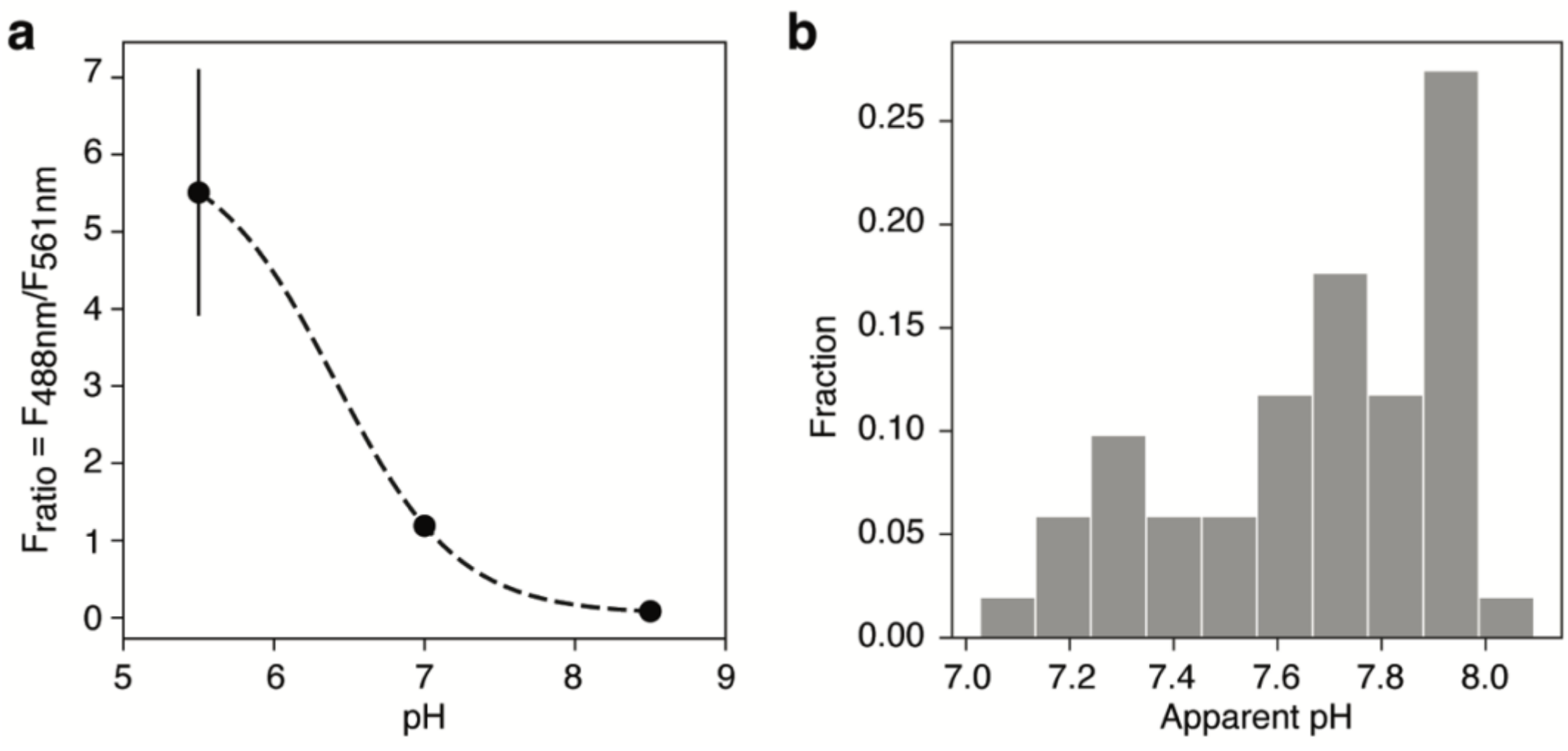

Supplementary Figure 9: CryoCLEM of pHmScarlet in *H. neapolitanus*.
a) F_{ratio} for 488- and 561-nm excitation of high-pressure frozen pHmScarlet as a function of pH using the same laser excitation power densities used for cryogenic cellular imaging. Each calibration point represents the average of at least 5 ratiometric measurements for each condition. Error bars represent the standard deviation. The dashed line is the fit to a continuous logistic function derived from the calibration in Fig. 2i. This was a control to ensure that the significantly different excitation intensities used for imaging did not change the calibration. **b)** Distribution of the apparent pH from single *H. neapolitanus* cells measured using cryogenic ratiometric imaging of pHmScarlet. *N* = 51 cells.

References

1. Cuevas-Velazquez, C. L. *et al.* Intrinsically disordered protein biosensor tracks the physical-chemical effects of osmotic stress on cells. *Nat Commun* **12**, 5438 (2021).
2. Tantama, M., Hung, Y. P. & Yellen, G. Imaging Intracellular pH in Live Cells with a Genetically Encoded Red Fluorescent Protein Sensor. *J. Am. Chem. Soc.* **133**, 10034–10037 (2011).
3. Linders, P. T. A., Ioannidis, M., ter Beest, M. & van den Bogaart, G. Fluorescence Lifetime Imaging of pH along the Secretory Pathway. *ACS Chem. Biol.* **17**, 240–251 (2022).
4. Ast, C. *et al.* Ratiometric Matryoshka biosensors from a nested cassette of green- and orange-emitting fluorescent proteins. *Nat Commun* **8**, 431 (2017).
5. Molina, R. S. *et al.* Understanding the Fluorescence Change in Red Genetically Encoded Calcium Ion Indicators. *Biophysical Journal* **116**, 1873–1886 (2019).
6. Stewart, S. A. *et al.* Lentivirus-delivered stable gene silencing by RNAi in primary cells. *RNA* **9**, 493–501 (2003).
